# In Situ Preparation of Silver Nanoparticles/Organophilic-Clay/Polyethylene Glycol Nanocomposites for the Reduction of Organic Pollutants

**DOI:** 10.3390/polym16243608

**Published:** 2024-12-23

**Authors:** Amina Sardi, Bouhadjar Boukoussa, Aouicha Benmaati, Kheira Chinoune, Adel Mokhtar, Mohammed Hachemaoui, Soumia Abdelkrim, Issam Ismail, Jibran Iqbal, Shashikant P. Patole, Gianluca Viscusi, Mohamed Abboud

**Affiliations:** 1 Département de Chimie Ouled Fares, Faculté Science Exacte Et Informatique, Université Hassiba Ben Bouali, Chlef 02010, Algeria; a.sardi@univ-chlef.dz; 2 Laboratoire de Chimie Physique Macromoléculaire L.C.P.M., Université Oran1 Ahmed Ben Bella, BP 1524, El-Mnaouer, Oran 31000, Algeria; 3 Laboratoire de Chimie des Matériaux L.C.M., Université Oran1 Ahmed Ben Bella, BP 1524, El-Mnaouer, Oran 31000, Algeria; bbouhdjer@yahoo.fr (B.B.); mohamedb.f@live.fr (M.H.); abdelkrim_soumia@yahoo.fr (S.A.); 4 Département de Génie des Matériaux, Faculté de Chimie, Université des Sciences et de la Technologie Mohamed Boudiaf, BP 1505, El-Mnaouer, Oran 31000, Algeria; 5 Laboratoire de Chimie Fine L.C.F., Université Oran1 Ahmed Ben Bella, BP-1524, El-Mnaouer, Oran 31000, Algeria; aouichabenmaati@yahoo.fr; 6 Ecole Nationale Polytechnique d’Oran Maurice Audin, ENPO-MA, BP-1523, El-Mnaouer, Oran 31000, Algeria; 7 Laboratoire Physico-Chimie des Matériaux-Catalyse et Environnement (LPCM-CE), Université des Sciences et de la Technologie d’Oran Mohamed Boudiaf (USTO-MB), BP 1505, El-Mnaouer, Oran 31000, Algeria; chinoune307@yahoo.fr; 8 Department of Process Engineering, Faculty of Science and Technology, University of Relizane, Relizane 48000, Algeria; 9 Département de Sciences de la Matière, Institut des Sciences et Technologies, Université Ahmed Zabana, Relizane 48000, Algeria; 10 Institut des Sciences et Techniques Appliquées (ISTA), Université Oran1 Ahmed Ben Bella, BP 1524, El-Mnaouer, Oran 31000, Algeria; 11 Department of Chemical Engineering, Khalifa University of Science and Technology, Abu Dhabi P.O. Box 127788, United Arab Emirates; isma0074@umn.edu; 12 Department of Environmental Sciences and Sustainability, College of Natural and Health Sciences, Zayed University, Abu Dhabi P.O. Box 144534, United Arab Emirates; jibran.iqbal@zu.ac.ae; 13 Department of Physics, Khalifa University of Science and Technology, Abu Dhabi P.O. Box 127788, United Arab Emirates; shashikant.patole@ku.ac.ae; 14 Department of Industrial Engineering, University of Salerno, Via Giovanni Paolo II, 132, 84084 Fisciano, SA, Italy; 15 Catalysis Research Group (CRG), Department of Chemistry, College of Science, King Khalid University, P.O. Box 9004, Abha 61413, Saudi Arabia; abboud_med@yahoo.fr

**Keywords:** organophilic clays, nanocomposite caly-polymer, dye reduction

## Abstract

This work focuses on the preparation and application of silver nanoparticles/organophilic clay/polyethylene glycol for the catalytic reduction of the contaminants methylene blue (MB) and 4-nitrophenol (4-NP) in a simple and binary system. Algerian clay was subjected to a series of treatments including acid treatment, ion exchange with the surfactant hexadecyltrimethylammonium bromide (HTABr), immobilization of polyethylene glycol polymer, and finally dispersion of AgNPs. The molecular weight of polyethylene glycol was varied (100, 200, and 4000) to study its effect on the stabilization of silver nanoparticles (AgNPs) and the catalytic activity of the resulting samples. The results showed that the catalyst with the highest molecular weight of polyethylene glycol had the highest AgNP content. Catalyst mass, NaBH_4_ concentration, and type of catalyst were shown to have a significant influence on the conversion and rate constant. The material with the highest silver nanoparticle content was identified as the optimal catalyst for the reduction of both pollutants. The measured rate constants for the reduction of methylene blue (MB) and 4-nitrophenol (4-NP) were 164 × 10^−4^ s^−1^ and 25 × 10^−4^ s^−1^, respectively. The reduction of MB and 4-NP in the binary system showed high selectivity for MB dye, with rate constants of 64 × 10^−4^ s^−1^ and 9 × 10^−4^ s^−1^ for MB and 4-NP, respectively. The reuse of the best catalyst via MB dye reduction for four cycles showed good results without loss of performance.

## 1. Introduction

The contamination of water sources by dyes represents a significant environmental concern, particularly in regions where industrial activities involving the use of chemicals for the dyeing of fabrics, leather, and paper products, as well as in the food and cosmetics industries, are prevalent [1,2,3,4]. The discharge of these dyes into rivers, lakes, and other bodies of water has resulted in adverse effects on the environment and human health [1,2,3,4]. The remediation of water contaminated by dyes is of paramount importance to mitigate the environmental impact and safeguard public health. Given that dyes are frequently synthetic, chemical, and resistant to degradation, their removal necessitates the deployment of suitable technologies [1,2,3,4]. A variety of techniques can be employed to effectively treat water contaminated by dyes. These include physical methods, such as adsorption, filtration, and flotation processes [5,6,7], as well as chemical methods based on oxidation reactions (advanced treatment AOP processes, ozone oxidation, photocatalysis), chemical precipitation, and chemical reduction [8,9,10,11]. Biological methods, on the other hand, rely on bioremediation, utilizing bacteria and fungi [12].

Among the aforementioned techniques, chemical reduction is particularly prevalent due to the pivotal role it plays in reducing chemical toxicity [13,14]. This is attributed to the fact that the by-products generated through this process are inherently less toxic [9]. Furthermore, the versatility of this method enables its utilization in a multitude of chemical reactions [15,16], thereby extending its relevance across diverse scientific and industrial domains. The dye reduction reaction involves the use of nanocatalysts that have the ability to transfer electrons from the donor (reducer) to the acceptor (dye) [13,14]. This process usually involves the use of reducing agents (such as hydrogen) or chemical reductants (such as sodium borohydride) to modify the chemical structure of the dyes [16].

Metallic nanoparticles based on Ag, Au, Pd, and Cu have been widely used in this field due to their catalytic performance in reducing dyes in shorter reaction times [17,18,19,20]. However, the aggregation problem of metal nanoparticles is a major challenge; hence, these particles have a great tendency to be agglomerated in the reaction medium, which can lead to a loss of catalytic efficiency.

It has been shown in several studies that the dispersion of nanoparticles in organic matrices (such as polymers) [21,22,23] or inorganic matrices (such as clays) [20,24,25,26] leads to the stability of nanoparticles; even their sizes can be reduced to ultrafine particles. It is for this reason that several studies have been conducted in this field of research. It has been shown that the use of clays as supports for metallic nanoparticles generates good properties via the reduction of organic pollutants [20,24,25,26]. But, the existence of metallic nanoparticles in clay galleries can sometimes lead to strong leaching of nanoparticles in the reaction medium.

According to the literature, it has been shown that the use of composite clay–polymer matrices can improve the stability of metal nanoparticles and good dispersions can be obtained [27,28,29,30]. This is related to the several interactions existing (physical or chemical) between metal nanoparticles and the functional groups existing in polymers [27,28,29,30]. The performance of the resulting catalyst can also be influenced by the nature of the polymer used (functional groups, its hydrophilic/hydrophobic character, the molecular mass of the polymer, its porosity, etc.) [27,28,29,30].

In this work, in terms of our research plan, a series of organophilic montmorillonite (MMT) clays modified by polyethylene glycol (PEG) with different molecular weights were used as catalytic supports for silver nanoparticles. The different nanocomposites were characterized by different methods in order to determine their structural, thermal, and catalytic properties. The nanocomposites were tested via the reduction of the methylene blue (MB) dye as a model reaction in an aqueous medium. The effect of molecular weight on the stabilization of AgNPs was studied in detail and the results were correlated according to the molecular weight of the polymer used, the surface charge, the AgNP content, their dispersions, as well as regarding their catalytic activity.

## 2. Experimental Section

### 2.1. Materials and Methods

The clay used in this study was obtained from the Hammam Boughrara deposit in Algeria. The surfactant hexadecyltrimethylammonium bromide (HTABr) (CH_3_(CH_2_)_15_N(Br)(CH_3_)_3_, 364.45 g/mol) was used for the swelling of the clay sheets. Polyethylene glycol PEG at different molecular weights (100, 200, and 4000) was used as a matrix for the stabilization of silver nanoparticles. Sodium tetrahydridoborate (NaBH_4_, CAS number 16940-66-2 and Mw 37.83 g/mol) was used as a reducing agent; organic pollutants methylene blue (C_16_H_18_ClN_3_S, CAS number 61-73-4 and Mw 319.85 g/mol) and 4-nitrophenol (O_2_NC_6_H_4_OH, CAS number 100-02-7 and Mw 139.11 g/mol) were purchased from Sigma-Aldrich (Sigma-Aldrich, St. Louis, MO, USA).

### 2.2. Preparation of Nanocomposites of AgNPs@Organophil-Clay

Nanocomposites containing organophilic MMT clay and PEG with different molecular weights (100, 200, and 4000) were prepared according to a well-detailed method [7]. This preparation method consists of a first step including acid treatment, followed by an ion exchange using the surfactant HTABr, and then the immobilization of the PEG polymer using ultrasonic treatment in each preparation step [31].

For the preparation of organophilic clay MMT-HTABr, 5 g of treated MMT was dispersed in a solution containing 100 mL of distilled water and 1 g of surfactant HTABr. The reaction mixture was stirred for 48 h at room temperature; at the end of the ion exchange process by surfactant HTABr, the resulting solid was washed by distilled water, filtered, and dried at 60 °C for 24 h. Then, for the preparation of MMT-HTABr-PEG nanocomposite, 1 g of organophilic clay (MMT-HTABr) was dispersed in 100 mL of distilled water, and then 0.5 g of PEG was used; the reaction mixture was treated by ultrasound for 1 h under an amplitude of 70%. The final product was washed with distilled water, filtered, and then dried at 60 °C for 24 h. The same protocol was used by simply varying the molecular mass of the PEG polymer (100, 200, and 4000).

The obtained nanocomposites at the end of the reaction were used as supports to prevent the aggregation of silver nanoparticles. For this, 1 g of nanocomposite was dispersed in 100 mL of an aqueous solution of 0.1 M AgNO_3_. To obtain a good dispersion of silver on the support, the reaction mixture was treated by sonication using an amplitude of 40% for 1 h. Then, the suspension was filtered and dried, and a solution of NaBH_4_ of concentration 1 M was added to reduce Ag^+^ species to zero-charge AgNPs; the mixture was stirred for 1 h with a speed of 600 rpm. At the end of the reaction, the product was recovered by washing using distilled water, and then by filtration and drying at 60 °C overnight. The same protocol was used for the preparation of the three nanocomposites; the different obtained catalysts were named as follows: Nano-1, Nano-2, and Nano-3 according to the PEG used, 100, 200, and 4000, respectively.

### 2.3. Reduction of Organic Pollutants in the Presence of Nanocomposites

In this work, we have studied various conditions that can affect the hydrogenation reaction of pollutants, such as the nature and the amount of nanocomposite and the concentration of NaBH_4_. The optimization of the operating conditions began by the reduction of an aqueous solution of methylene blue (MB) with an initial concentration of 0.1 mM in the presence of different amounts of nanocomposite. In a quartz tank, we put the nanocomposite (1 mg, 2 mg, and 3 mg) with 2 mL of methylene blue solution. Well-defined concentrations of freshly prepared NaBH_4_ (10–20 mM), with a volume equal to 1 mL, were added individually to the initial solution. The reaction progress was followed by spectrophotometry UV–vis. The reduction of methylene blue (MB) was determined by analyzing the decrease in maximum absorbance (λmax) at 664 nm until its disappearance. The solution recovered at the end of the reaction was colorless, which proves a total reduction of methylene blue (MB) to leuco-methylene blue (Leuco-MB).

The pH of the solution was not adjusted, but the pH was changed during the mixing with NaBH_4_ in the reaction medium from where it was 12 and 10 for MB and 4-NP, respectively.

The values of dye conversion and the rate constant kapp (s^−1^) were calculated using the appropriate equations (Equations (1) and (2), respectively). In this context, *C*_0_ and *C_t_* refer to the initial and final dye concentrations, respectively, and kapp (s^−1^) is the rate constant.

(1) $$Dye\;conversion\;\left(\%\right)=\frac{C_{0}-C_{t}}{C_{0}}\times 100\%$$

(2) $$Ln\;\frac{C_{t}}{C_{0}}=-K_{app}\times t$$

### 2.4. Characterization of Materials

The structural properties of different nanocomposites were determined by X-ray diffraction (XRD) using a Bruker D8 powder diffractometer (Cu-Kα radiation; wavelength λ = 1.5406 Å) (Bruker, Billerica, MA, USA). The nanoparticle size, shape, and dispersion degree were determined by Titan 80-300 ST transmission electron microscope TEM (Thermo Fisher Scientific Inc., Waltham, MA, USA). The content of silver nanoparticles in each sample was determined by X-ray Fluorescence (XRF) using X-MET8000 instrument (Hitachi High-Tech, Tokyo, Japan). The different functional groups existing in the nanocomposites were determined by Fourier Transform Infrared (FTIR) spectroscopy using a JASCO 4100 Spectrometer (JASCO Corporation, Tokyo, Japan). The surface charge of the nanocomposite at different pH was determined by zeta potential using the Zetasizer (model Nano-ZS, Malvern Instruments, England, UK). X-ray photoelectron spectroscopy (XPS) was employed to ascertain the elemental composition of the synthesized nanocomposites, utilizing a Thermo Scientific ESCALAB Xi+ spectrometer (Thermo Fisher Scientific Inc., Waltham, MA, USA). The catalytic reduction of methylene blue (MB) and 4-nitrophenol (4-NP) was assessed through UV–vis spectroscopy using a Speccord 210 (Analytik Jena, Jena, Germany).

## 3. Results

### 3.1. Characterization of Nanocomposites

#### 3.1.1. XRD/XRF

**Figure 1:**
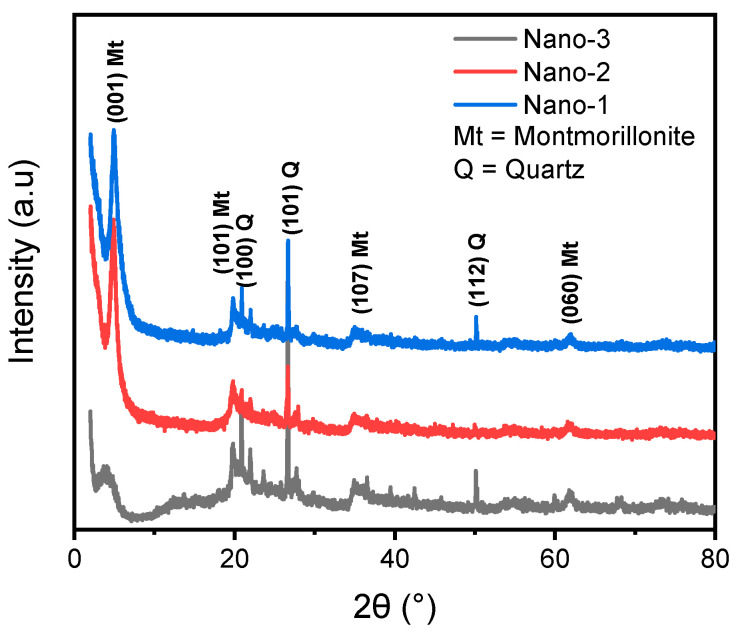
XRD patterns of obtained Nano-1, Nano-2, and Nano-3 nanocomposites.

The powder XRD patterns of the Nano-1, Nano-2, and Nano-3 samples are shown in Figure 1. All the nanocomposites have (001), (101), (107), and (060) reflection planes corresponding to the structure of Maghnia montmorillonite [31,32,33]. According to the XRD spectra of the different samples, the quartz is still present despite the clay already having been treated with acid; it is characterized by the following planes: (100), (101), and (112) [33].

According to previously published work, the raw Maghnia clay has an interfoliar distance of 1.01 nm, and its Na^+^ exchanged form has an interlayer distance of 1.26 nm [6]. The calculations of the interlayer distance based on the (001) reflection led to the following results: 1.80, 1.88, and 2.3 nm for nanocomposites Nano-1, Nano-2, and Nano-3, respectively. This increase in interfoliar distance is strongly due to the intercalation of HTABr, PEG, and AgNPs in the clay galleries. It is noteworthy that the basal spacing increases significantly with increasing PEG molecular weight; hence, PEG(4000)-intercalated Nano-3 exhibited the largest interfoliar distance compared to the other materials. However, no characteristic peaks of silver nanoparticles were observed in the XRD patterns. This is due to the low content of AgNPs and also to their covering by the clay layers and the PEG polymer. To confirm this finding, an XRF analysis was carried out, demonstrating that the percentages of AgNPs were 0.9%, 1%, and 1.8% for the Nano-1, Nano-2, and Nano-3 samples, respectively. It is clear that there is a direct relationship between mass and content of AgNPs: as the molar mass of PEG increases, the content of AgNPs increases. This also provides a general idea about the role of the PEG polymer in retaining silver nanoparticles through multiple interactions between the functional groups of PEG and AgNPs.

The increase in silver nanoparticle (AgNP) content with higher-molar-mass polyethylene glycol (PEG) is not solely due to the number of oxygen-containing groups as the proportion of oxygen-containing groups per unit mass decreases with increasing molar mass. Instead, it can be attributed to the physical and chemical properties of PEG. Higher-molar-mass PEG provides greater steric stabilization, creating a robust barrier around AgNPs, which prevents agglomeration and enhances dispersion [34,35]. Additionally, longer PEG chains exhibit a higher probability of entanglement and interaction with the AgNP surfaces, thereby stabilizing their formation [36]. The higher viscosity of larger PEG molecules also influences the reaction kinetics during silver ion reduction, promoting effective nucleation and growth of AgNPs [37]. Furthermore, the spatial distribution of the longer chains facilitates efficient coverage of the nanoparticle surface, resulting in a higher overall AgNP content. These combined steric, kinetic, and adsorption effects explain the observed increase despite the constant mass content of PEG in the composites.

#### 3.1.2. FTIR

**Figure 2:**
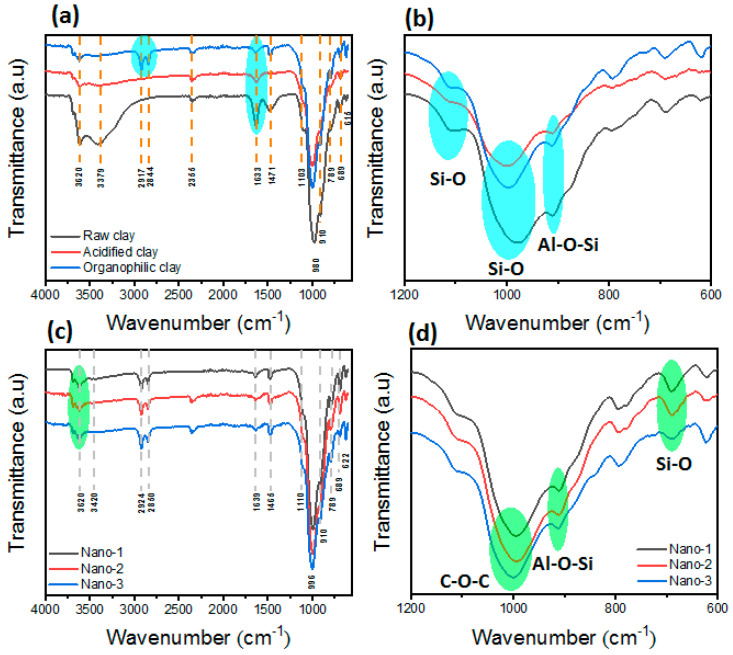
FTIR spectra of obtained samples before and after modification.

The FTIR spectra of raw, acidified, and organophilic clay containing HTABr show almost the same bands but with a slight change due to acid treatment and ion exchange by HTABr. As shown in Figure 2a,b, all three materials exhibit an average band located at 3620 cm^−1^, mainly due to the presence of the stretched O–H bond [31]. The broad band at 3379 cm^−1^ corresponds to the vibrations of the H–O–H stretching bond, mainly due to the water molecules intercalated through hydrogen bonds coming from the terminal silanol groups of the aluminosilicate layer [38]. The presence of a weak band at 1471 cm^−1^ corresponds to the vibrations of CO_3_ stretching, confirming the presence of traces of carbonate impurities [39]. At 1635 cm^−1^, a medium band is observed due to the asymmetric bending mode of the intercalated water [31]. It is clear that the bands located at 3379 cm^−1^ and 1635 cm^−1^ were decreased after the treatment due to the presence of HTABr intercalated in the clay galleries [31]. This was also confirmed by the presence of the new bands for HTABr-modified clay at about 2917 cm^−1^ and 2844 cm^−1^ corresponding to the symmetric and asymmetric stretching vibrations of the CH_2_ group of surfactant HTABr [31]. The weak band located around 1103 cm^−1^ is due to the presence of the trivalent Al^3+^ and Fe^3+^ tetrahedral ions substituted by silicon in octahedral localizations of clay [39]. The intense band observed around 980 cm^−1^ is assigned to the stretching vibrations of the Si–O bond (of a tetrahedral structure) [40]. After the intercalation of HTABr, this band was moved towards the highest wavenumbers from 980 to 996 cm^−1^, which is due to the multiple interactions between the surfactant and the clay layers [31]. The band observed at 910 cm^−1^ is mainly due to the vibrations of the Al–O–Si bond, which are attributed to montmorillonite [31]. The weak band appearing at 789 cm^−1^ is characteristic of the Si–O stretching of quartz [38]. It should be noted that the FTIR spectra of AgNPs–PEG–organophilic clay nanocomposites have almost the same absorption bands as organophilic clay due to the overlapping between them (see Figure 2c,d) [31]. For example, the vibration bands of –CH_2_ corresponding to PEG have the same vibrations as surfactant containing clay (the bands located around 2924 and 2850 cm^−1^) but with a slight shift towards higher wavenumbers. The bands located around 1110 cm^−1^ and 996 cm^−1^ correspond to the C–C + C–O–C and CH_2_ + C–O–C stretching vibrations of PEG [41], which overlap with the bands of Si–O [38]. It is clear that the OH binding of PEG also overlaps with those of organophilic clay (the bands located around 3420 and 1639 cm^−1^). However, a shift towards higher wavenumbers was observed compared to organophilic clay. This is strongly related to the strong interactions between the silver nanoparticles AgNPs, which is in agreement with the XPS results.

#### 3.1.3. XPS

**Figure 3:**
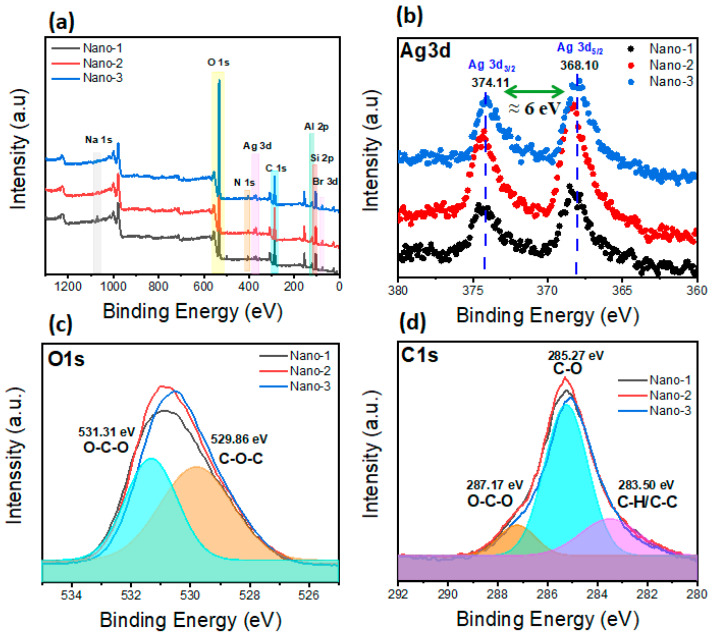
XPS spectra of different nanocomposites: (**a**) XPS survey spectra, (**b**) high-resolution Ag3d XPS, (**c**) high-resolution O1s XPS, and (**d**) high-resolution C1s XPS.

XPS analysis was used for the determination of the type of species present in the different samples as well as their oxidation states. As indicated via the XPS survey spectra (Figure 3a), all the samples show the same elements. The presence of the peaks of N1s, C1s, and Br3d confirms the presence of the surfactant HTABr, whereas Na1s, Si2p, O1s, Na1s, and Al2P are the main clay constituents. Meanwhile, for the PEG polymer, its peaks overlap with those of HTABr and clay (in the cases of O1s and C1s). The presence of the Ag3d peak confirms the presence of silver on the nanocomposite surface. The XPS spectra of Ag3d (Figure 3b) for the different samples show two peaks around 368.10 eV and 374.11 eV, corresponding to Ag 3d_5/2_ and Ag 3d_3/2_, respectively [42,43]. It is noticed that these spectra are shifted weakly towards higher binding energies due to the strong interactions between the silver nanoparticles (AgNPs) and hydroxyl groups containing the clay and PEG [42,43]. Overall, the energy variation regarding these two peaks is around 6 eV for all the samples, confirming the formation of zero-charge silver nanoparticles [42,43]. This result confirms that the silver nanoparticles are well formed on the surface of the nanocomposite. These results are in agreement with the binding energy of O1s (Figure 3c), where two sub-peaks were observed at 531.31 eV and 529.86 eV corresponding to the O-C-O and C-O-C bonds, respectively. These peaks have shifted weakly to a higher binding energy compared to the literature, mainly due to the decreasing electron density of O atoms following the multiple interactions between AgNPs and oxygen [43]. In addition, the C1s spectra of different nanocomposites showed three binding energy peaks around 287.17 eV, 285.27 eV, and 283.50 eV, corresponding, respectively, to the O-C-O, C-O, and C-H/C-C bonds (Figure 3d). These peaks were also shifted weakly to higher binding energies, which confirms that the electron density of the C atoms was influenced by C-O interaction in the nanocomposites and silver nanoparticles [44].

#### 3.1.4. TGA

**Figure 4:**
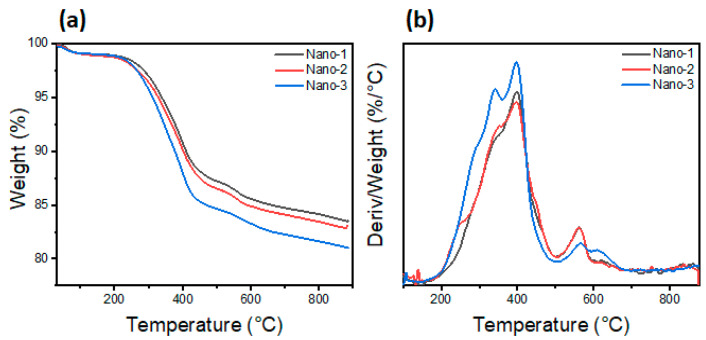
Thermal analysis of different samples: (**a**) TGA curves; (**b**) DTG curves.

The thermal analysis of different samples is shown in Figure 4. From this figure, all the samples show almost the same curve but with a slight variation in mass loss. According to the TGA and DTG curves, all the samples show a slight loss of mass around 80 °C due to the desorption of water molecules physically adsorbed on their surface. The second mass loss starts at 300 °C, accompanied by another slight mass loss at 560 °C, corresponding to the degradation of the organic matter (surfactant HTABr and PEG). The mass losses obtained in this temperature range are as follows: 12%, 13%, and 15% for nanocomposites Nano-1, Nano-2, and Nano-3, respectively. Clearly, the thermal stability of different samples is inversely related to PEG molecular weight. The stability of nanocomposites increases slightly with decreasing PEG molecular weight.

#### 3.1.5. TEM

**Figure 5:**
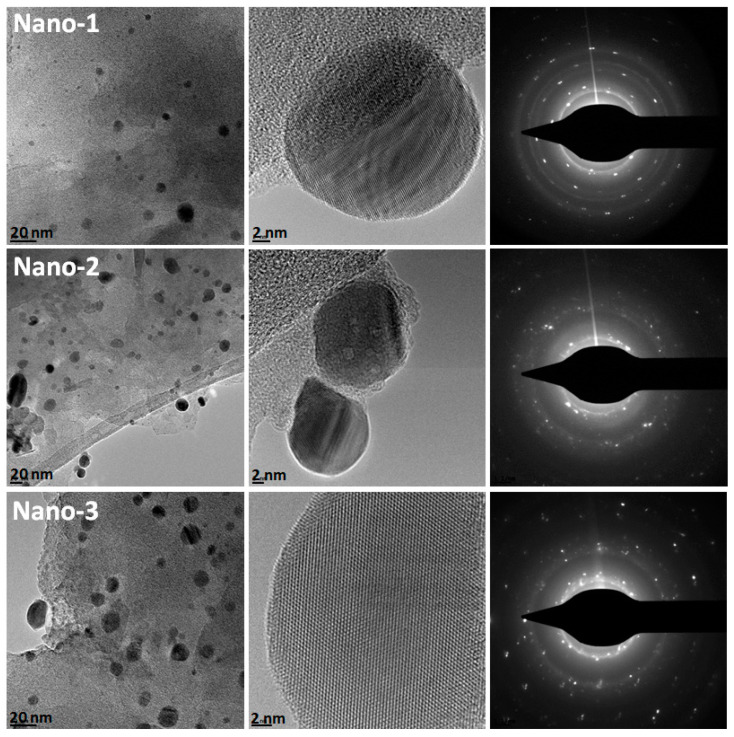
TEM images of obtained Nano-1, Nano-2, and Nano-3 nanocomposites.

The silver nanoparticles supported on the PEG–organophilic clay were observed by TEM analysis. As shown in Figure 5, the silver nanoparticles have a spherical shape with good dispersion on the support, and no agglomeration phenomenon was observed, which shows the beneficial effect of ultrasonic treatment. From this figure, it is clear that the molecular weight of PEG plays a very important role via the dispersion and retention of AgNPs. The Nano-3 material exhibited the highest density of AgNPs with a high degree of dispersion due to the multiple interactions between the AgNPs and the functional group of PEG, which is in agreement with the XPS analysis. The particle size of AgNPs is about 5 to 24 nm, with an average size of about 9 nm, as determined from the TEM images of the Nano-2 sample. Furthermore, the lattice fringes of AgNPs exhibit a spacing of 0.227 nm, corresponding to the (111) crystallographic plane of face-centered cubic (FCC) silver nanoparticles [45]. The SAED images show polycrystalline diffraction rings of metallic silver nanoparticles, which is in agreement with previously published work [46].

### 3.2. Reduction of Organic Pollutants

#### 3.2.1. Effect of Catalyst Mass

The reduction of organic pollutants has been considered as an effective strategy for the degradation of pollutants like dyes, nitrophenolic derivatives, and transition metals because this treatment process not only decreases the toxicity but the products formed can also be used in several fields, particularly in organic synthesis [47]. The advantages of this reaction also lie in the rapidity of this process compared to other reactions [48,49,50]. It should be noted that the simplicity has also widened the application field of this reaction, with even toxic transition metals such as Cr(VI) being reduced to Cr(III), which is impossible by other treatment processes. MB dye reduction is one of the model reactions to test the efficiency of a catalyst [51,52,53]. For this reason, we have chosen this reaction by varying some parameters.

**Figure 6:**
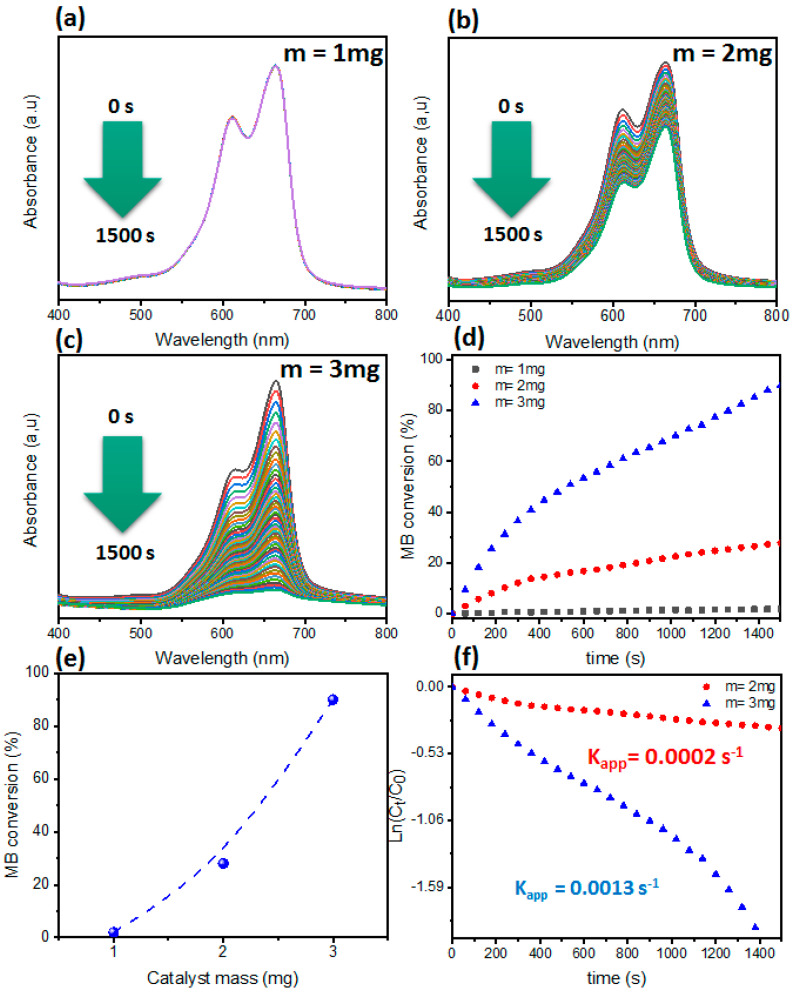
(**a**–**c**) UV–vis of MB dye catalyzed by Nano-1 at different masses. (**d**) Conversion of MB dye as a function of time. (**e**) Correlation plot between Nano-1 catalyst mass and MB dye conversion. (**f**) Plot of ln(C_t_/C_0_) as a function of time.

Firstly, the effect of catalyst mass was studied by varying the mass of the Nano-1 catalyst between 1 and 3 mg while keeping the other conditions constant. From Figure 6, it is clear that there was a direct relationship between the catalyst mass and the MB dye conversion. It should be noted first that, without a catalyst, no reaction progress was observed even at a 20 mM NaBH_4_ concentration [17,47]. Then, from the addition of low catalyst mass, the characteristic methylene blue band located at around 664 nm slowly decreased. According to the conversion curve, the reduction of MB dye was approximately 90% in a reaction time of 70 min. Then, on increasing the mass to 2 and 3 mg, the MB conversion was about 92% and 96%, recorded in reaction times of 60 and 30 min, respectively. The calculated rate constants under these conditions were 2 × 10^−4^ s^−1^ and 13 × 10^−4^ s^−1^ during the use of catalyst masses of 2 mg and 3 mg, respectively. This also demonstrates the beneficial effect of the catalyst. From the literature, it has been confirmed that an increase in catalyst mass leads to an increase in the content of the active sites (AgNPs) responsible for the reduction of MB dye [47,54]. Their roles are to transport electrons from the donor to the acceptor [47,54].

#### 3.2.2. Effect of NaBH_4_ Concentration

**Figure 7:**
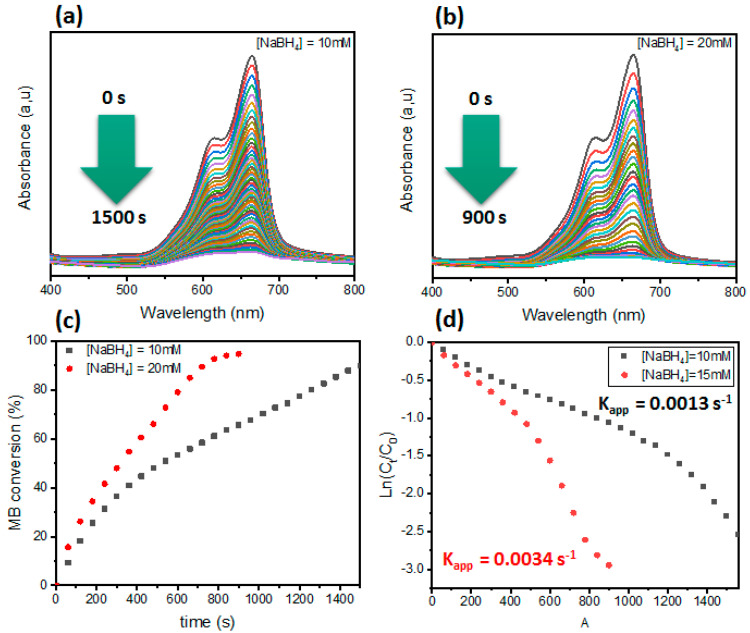
(**a**,**b**) UV–vis of MB dye catalyzed by Nano-1 catalyst at different concentrations of [NaBH_4_]. (**c**) Conversion of MB dye as a function of time. (**d**) Plot of Ln(C_t_/C_0_) as a function of time.

The effect of NaBH_4_ concentration plays a crucial role in the reduction reaction. For this reason, we also varied the NaBH_4_ concentration between 10 and 20 mM (Figure 7). First, a control reaction without a catalyst was tested using a 20 mM NaBH_4_ concentration, where the results confirmed that, without a catalyst, no reaction progress was observed even if the reaction time was prolonged (see Supplementary File Figure S1). It should also be taken into consideration that the presence of the Nano-1 material alone does not show any adsorption affinity for MB dye (see Supplementary File Figure S2). So, when using the Nano-1 catalyst with 10 mM NaBH_4_, the reaction time was around 1500 s, but, when increasing the concentration of NaBH_4_ to 20 mM, the reduction was rapid; hence, the MB conversion was complete and the reaction time was approximately 900 s, twice lower compared to that of 10 mM. The rate constant was improved to 34 × 10^−4^ s^−1^ when using a high concentration of NaBH_4_ (20 mM). According to the literature, increasing the concentration of NaBH_4_ leads to the formation of an electronic layer on the surface and also a high content of active hydrogen species, which can accelerate the reaction rate. These results are in agreement with those obtained in the literature [55].

#### 3.2.3. Effect of Catalyst Nature

**Figure 8:**
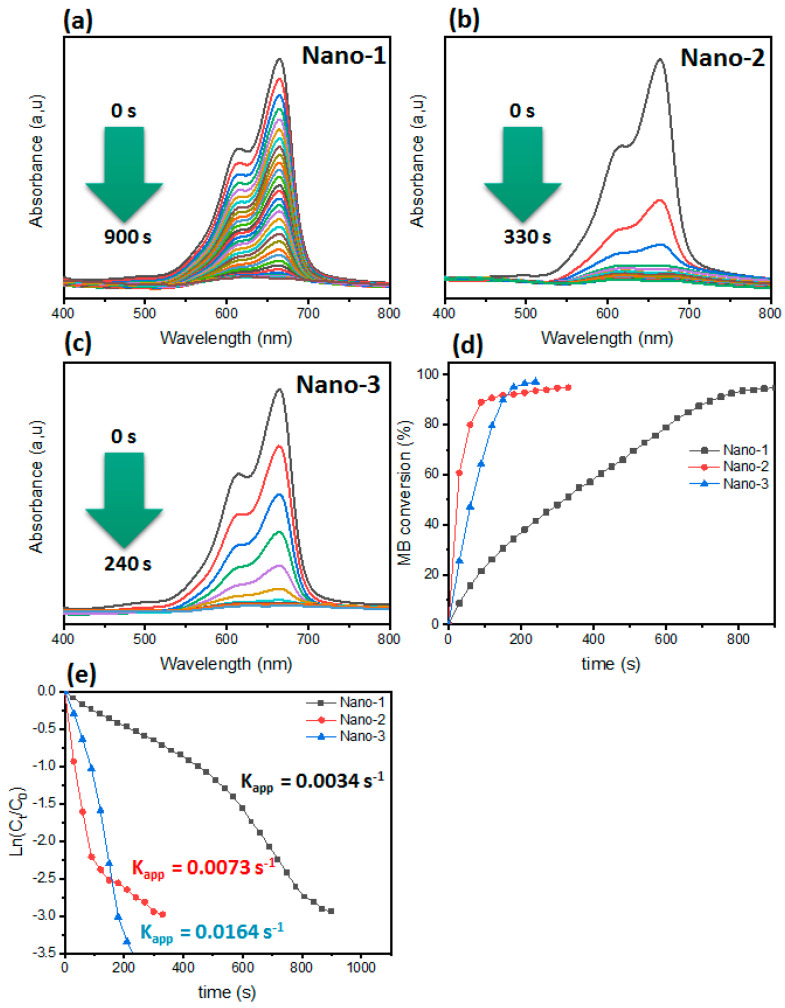
(**a**–**c**) UV–vis of MB dye catalyzed by different catalysts. (**d**) Conversion of MB dye as a function of time. (**e**) Plot of Ln(C_t_/C_0_) as a function of time.

As shown in Figure 8, all the UV–vis spectra show a reduction evolution regarding MB dye. It is clear that the characteristic band of MB dye located around 664 nm decreased during the reaction. The reaction time differs from one catalyst to another, which confirms that their surface compositions are different. According to Figure 8, the MB conversion was complete after a reaction time of 240 s for the Nano-3 catalyst. In all the cases, this catalyst was the most efficient and the fastest in reducing all the MB dyes. The rate constants recorded for the Nano-1, Nano-2, and Nano-3 catalysts were 34 × 10^−4^ s^−1^, 73 × 10^−4^ s^−1^, and 164 × 10^−4^ s^−1^, respectively. According to the previously determined physicochemical properties, all the catalysts showed almost the same structural and thermal properties, but their morphologies and chemical compositions differed. The material Nano-3 showed a good dispersion of AgNPs. The content of AgNPs is high in the Nano-3 catalyst, which enables it to achieve good catalytic performance via MB dye reduction. From the literature, it has been found that dye reduction is better regarding catalysts with high densities of metal nanoparticles [47]. Their role is to transport electrons from donor to acceptor and also to form active hydrogen species. The comparison of this material (Nano-3) with the materials used in the literature showed encouraging results, particularly from economic and ecological points of view [56].

#### 3.2.4. Catalytic Reduction of MB Dye and 4-NP in a Binary System

The reduction of organic pollutants in multi-pollutant systems is considered to be the most demanded reaction since it is possible to have polluted waters with multiple contaminants. Accordingly, we chose to examine the reduction of MB dye and 4-NP in a binary system as a model reaction. Before examining this reaction, it is essential to evaluate the efficacy of the Nano-3 catalyst in reducing 4-NP in a simple system (see Figure S3). Numerous studies have demonstrated that the simultaneous presence of NaBH_4_ and 4-nitrophenol results in the formation of 4-nitrophenolate ions due to the alkalinity of the medium [57,58,59]. Furthermore, no reaction occurs regarding the production of 4-aminophenol, even at high concentrations of NaBH_4_ [57,58,59]. Upon the addition of the Nano-3 catalyst, a decrease in the band located around 400 nm was observed following its reduction to 4-aminophenol. The reaction time and rate constant were 390 s and 88 × 10^−4^ s^−1^, respectively, to achieve 85% conversion of 4-NP. These results are very promising compared to previously published works [60,61].

**Figure 9:**
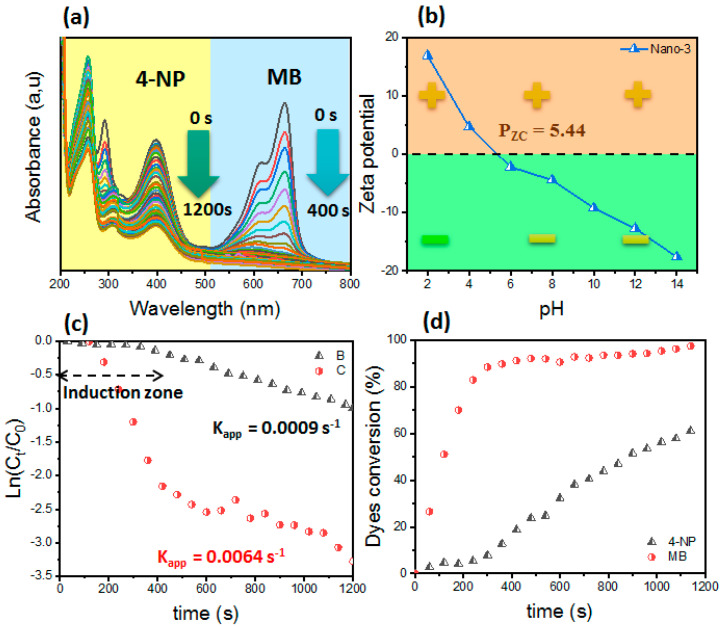
(**a**) UV–vis of MB dye and 4-NP catalyzed by Nano-3 catalyst in binary system. (**b**) Zeta potential as a function of solution pH. (**c**) Conversion of MB dye and 4-NP as a function of time. (**d**) Plot of ln(C_t_/C_0_) as a function of time.

For the binary system containing MB dye and 4-NP, the results are shown in Figure 9. The Nano-3 catalyst shows catalytic activity via both pollutants but with good selectivity via MB dye. It should be noted that the reduction of MB dye was complete in a reaction time of 400 s. Meanwhile, for the case of 4-NP, its reduction was partial; hence, about 60% of the 4-NP was reduced to 4-AP in a reaction time of 1200 s.

It should be taken into account that, after the total reduction of the MB dye, the 4-NP started its reduction (Figure 9). As shown by the induction zone for the pollutant 4-NP, its reduction will take place after the reduction of MB. This also confirms that there is an affinity between MB and the catalyst surface. From zeta potential measurements, it was found that the catalyst presents a negatively charged surface at basic pHs. According to the literature, the addition of NaBH_4_ in an aqueous solution leads to the formation of a basic solution. Therefore, in a basic solution, the cationic dye MB forms electrostatic attractions with the catalyst, which makes the adsorption of MB favorable compared to 4-NP [14]. Then, for the case of 4-NP, the latter transforms into 4-nitrophenolate ions, which form repulsive forces with the catalyst. Only van der Waals or hydrogen bonding can exist, which makes its adsorption slow compared to MB dye. For this reason, the reduction of 4-NP will take place after the conversion of MB dye. The rate constants recorded for 4-NP and MB are 9 × 10^−4^ s^−1^ and 64 × 10^−4^ s^−1^, respectively.

#### 3.2.5. Reuse of Nano-3 Catalyst

**Figure 10:**
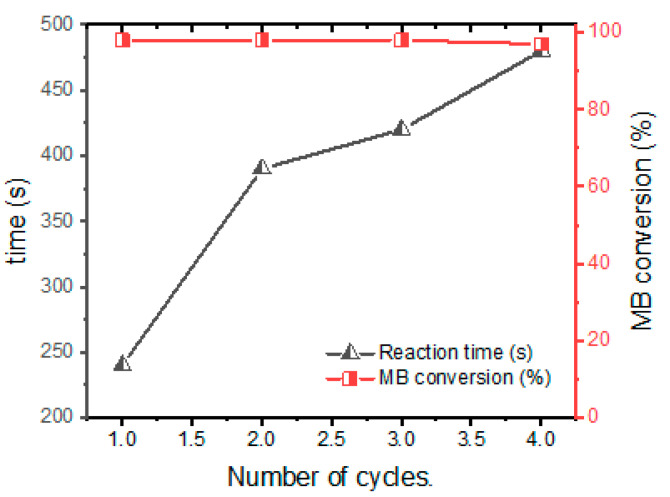
Reuse of Nano-3 catalyst via MB dye reduction.

The reuse of our best catalyst was investigated to study its stability in several cycles. For this, the Nano-3 catalyst was used to catalyze the MB dye reduction. In each reuse, the catalyst was just washed with distilled water and reused in a new cycle. The reuse results are shown in Figure 10. It is clear that this material presents a good catalytic performance via the reduction of MB dye as the conversion of MB dye is total in each cycle. This performance is due to the stability of the catalyst in the reaction medium. According to AgNP leaching tests, this catalyst was stable in each reuse, which enabled it to perform well in each reuse.

#### 3.2.6. Reduction Mechanism

Based on the results obtained, it is possible to propose a reduction mechanism. According to Figure S4, the starting reagent adsorbs on the catalyst’s surface. As shown previously, the adsorption of MB dye and 4-NP occurs differently. Based on zeta potential measurements, the most dominant interactions are electrostatic attractions between MB dye and the surface that is negatively charged, which increases the adsorption of MB relative to 4-NP. Then, in the case of 4-NP, it forms the 4-nitrophenolate ion due to the basicity of NaBH_4_ in the reaction medium, subsequently leading to hydrogen-type interactions or van der Waals bonds, which slow the adsorption of 4-NP. This is why the conversion of MB dye is faster than 4-NP in both studied systems. During the same adsorption period, the dissociation of NaBH_4_ results in the formation of hydrogen, which subsequently adsorbs on the surface of the catalyst [62,63]. Afterward, the hydrogen dissociates, subsequently yielding active hydrogen species. These hydrogen species bind to the pollutants, leading to a hydrogenation reaction [64]. For example, the reduction of 4-NP has several steps, leading subsequently to the formation of several unstable intermediates [65]. In general, this reaction requires six electrons and six hydrogen species to reduce 4-nitrophenol to 4-aminophenol [65]. The product formed, 4-aminophenol, is characterized by the presence of a new band around 300 nm (see Figure S3).

So, for the case of MB dye, the electrons and active hydrogen species coming from NaBH4 target the –N=C– and –C=N^+^(CH_3_)_2_ bonds to reduce them to –NH–CH– and –CH –HN^+^(CH_3_)_2_ [66]. The product formed from the reduction of the MB dye is Leuco-MB, which is characterized by the presence of a new band located around 259 nm (see Figure S5).

## 4. Conclusions

Organophilic clay–PEG–AgNP nanocomposites were well prepared using ultrasonic radiation. According to the TEM analysis, microporous and ultrafine nanoparticles were obtained with sizes ranging from 7 to 13 nm with a high degree of dispersion observed for the Nano-3 catalyst. According to the XRF analysis, the AgNP content varied in the following order: 0.9%, 1%, and 1.8% for the Nano-1, Nano-2, and Nano-3 nanocomposites, respectively. Increasing the molecular weight of PEG not only increased the AgNP content but also led to the formation of more stable AgNPs due to the multiple interactions between them. The XPS analysis confirmed the good formation of AgNPs without any formation of other species in the form of oxides. The application of these nanocomposites to the reduction of MB dye and 4-NP showed good results via MB dye in both single and binary systems. The best catalyst was the nanocomposite Nano-3 containing the higher content of AgNPs in its structure. It was shown that the reduction of MB dye in the single system was faster, where the total MB dye was reduced in 240 s, while, for 4-NP, the reaction time was 390s to achieve 90% conversion of 4-nitrophenol (24-NP) to aminophenol (4-AP). In the binary system, the catalyst Nano-3 was more selective via MB dye. This selectivity was expressed by the electrostatic attractions between the cationic MB dye and the negatively charged surface, as demonstrated by the zeta potential measurements. The reuse of the catalyst Nano-3 showed impressive MB conversion in four consecutive reuses, which shows its stability.

## Data Availability

No data were used for the research described in the article.

## Supplementary Materials

The following supporting information can be downloaded at https://www.mdpi.com/article/10.3390/polym16243608/s1, Figure S1: UV-vis spectrum of MB dye during the blank test. Figure S2: UV-vis spectrum of MB dye during adsorption test (adsorbent = Nano-1). Figure S3: (a) UV-vis spectrum of 4-NP during its reduction catalyzed by Nano-3. (b) Conversion curve of 4-NP and plot of Ln(Ct/C0) versus time. Figure S4: Mechanism for reduction of MB dye, and 4-NP. Figure S5: UV-vis spectrum of MB before and after reduction reaction using catalyst Nano-3.
