# Supplementary material for: In Situ Preparation of Silver Nanoparticles/Organophilic-Clay/Polyethylene Glycol Nanocomposites for the Reduction of Organic Pollutants
Source: Polymers (Basel). 2024 Dec 23;16(24):3608. doi: 10.3390/polym16243608 (PMC11679098; doi:10.3390/polym16243608)
Supplement: Supplementary file 1 [file polymers-16-03608-s001.zip › polymers-3339912-supplementary.pdf]

Supplementary material of

In Situ Preparation of Silver Nanoparticles/Organophilic-Clay/Polyethylene Glycol Nanocomposites for the Reduction of Organic Pollutants

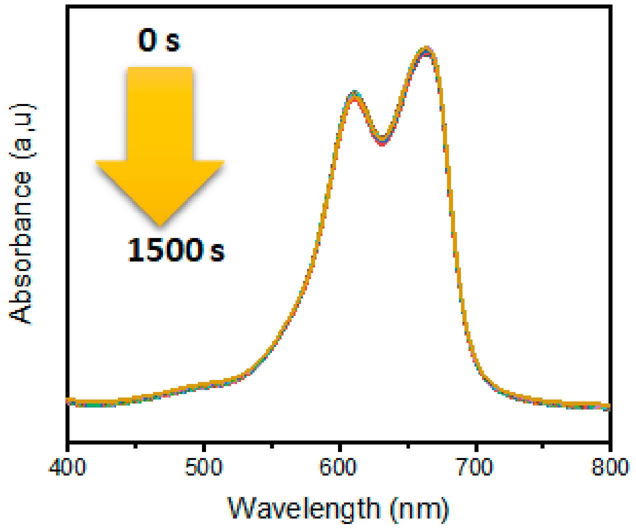

Figure S1. UV-vis spectrum of MB dye during the blank test.

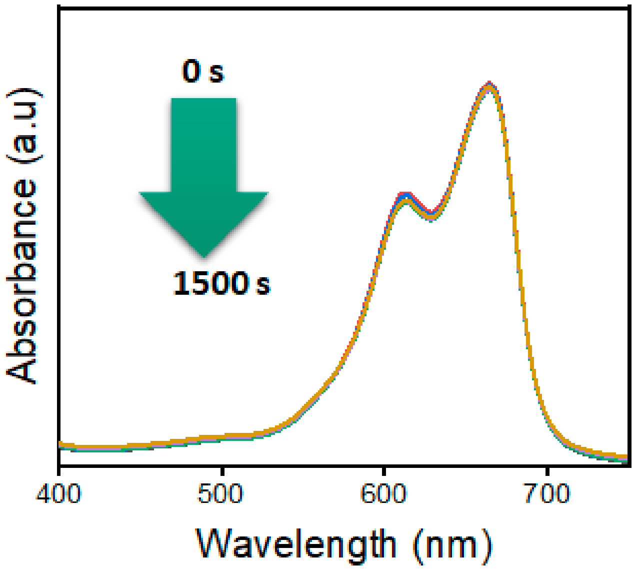

Figure S2. UV-vis spectrum of MB dye during adsorption test (adsorbent = Nano-1).

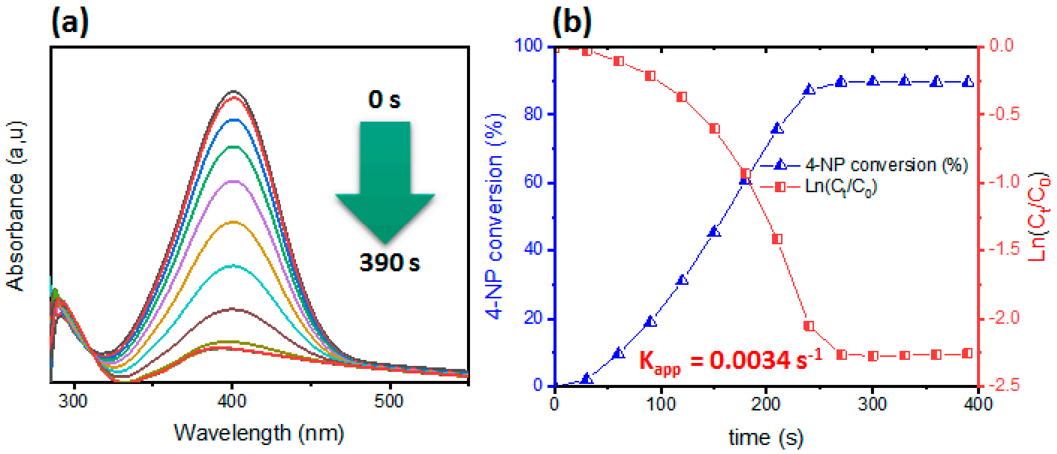

Figure S3. (a) UV-vis spectrum of 4-NP during its reduction catalyzed by Nano-3. (b) Conversion curve of 4-NP and plot of $Ln(C_t/C_0)$ versus time.

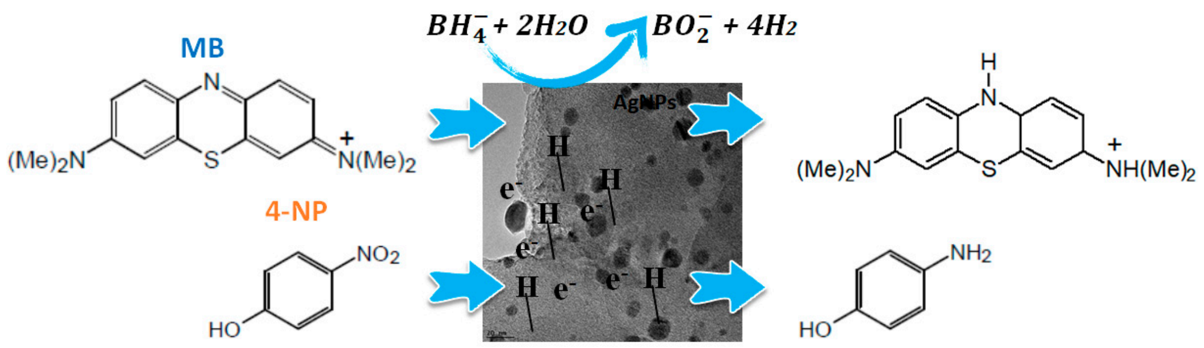

Figure S4. Mechanism for reduction of MB dye, and 4-NP.

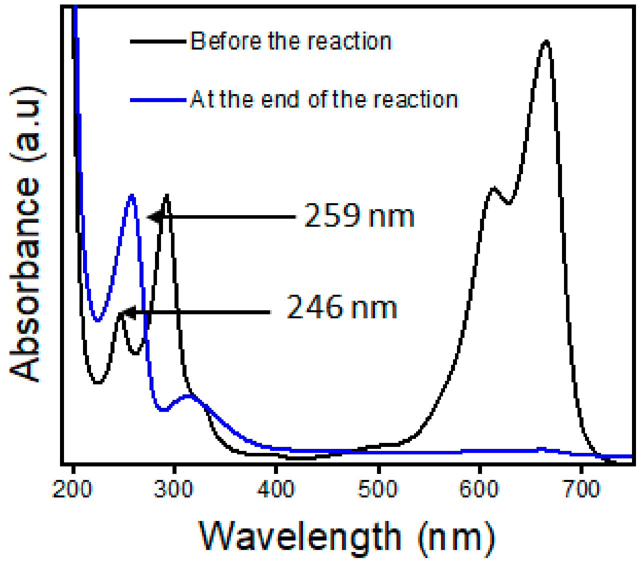

Figure S5. UV-vis spectrum of MB before and after reduction reaction using catalyst Nano-3.
